# Supplementary material for: A DNA/HDAC dual-targeting drug CY190602 with significantly enhanced anticancer potency
Source: EMBO Mol Med. 2015 Mar 10;7(4):438–49. doi: 10.15252/emmm.201404580 (PMC4403045; doi:10.15252/emmm.201404580)
Supplement: Supplementary file 1 — Supplementary Figures and Tables [file emmm0007-0438-sd1.pdf]

Supplementary information

Table of contents

1. **Supplementary Tables**

ⅰ.**Table *S1*** List of DNA repair genes analyzed by qPCR.

ⅱ.**Table *S2*** List of the qPCR primers for the analyzed DNA repair genes.

ⅲ.**Table *S3*** List of shRNA target sites of indicated genes.

ⅳ.**Table *S4*** List of qPCR primers used for analyzing shRNA knockdown efficiency.

2. **Supplementary Figures and Legends**

ⅰ. **Figure *S1*** HDAC inhibition by CY and Cpd A, but not bendamustine in H1650 cells. The HDAC inhibitor SAHA were used as positive control. Cells were treated at indicated drug concentration for 8hrs before collecting samples.

ⅱ. **Figure *S2*** Dose response curve of bendamustine, CY, Cpd A and Cpd B in H1650 cells. Cells were treated for 48hrs before MTT assay.

ⅲ. **Figure *S3*** ShRNA mediated suppression of Cbp at protein level.

ⅳ. **Figure *S4*** Suppression of CBP, Tip60, MORF and MSL1 did not affect cell viability without drug treatment. Data represent mean±SEM, from three independent experiments.

v. **Figure *S5*** HDAC inhibition resulted in suppression of EP300 and TYMS. Cells were treated for 6hrs before mRNA collection and qPCR analysis. Data represent mean±SEM, from three independent experiments. *p < 0.1, **p < 0.05, ***p < 0.01.

vi. Figure *S6* Suppression of EP300 and TYMS caused reduced viability of cancer cells. Retrovirus that encodes GFP and indicated shRNAs were used to infect cells to achieve 30-60% infection rate, and percentages of GFP positive, shRNA-expressing cells were followed

overtime. shRNAs that significantly impairs cell growth such as sh EP300 and sh TYMS showed rapid loss of GFP positive cells overtime.

Table *S1* List of DNA repair genes analyzed by qPCR.

ATR-CHK1 SIGNALING	FA pathway	TLS pathway	HR pathway	Nucleotide synthesis and others
ATR	FAN1	POLI	ATM	ADSS
ATRIP	FANCA	POLK	BLM	APEX1
CBP	FANCB	POLN	BRCA1	CTPS
CHK1	FANCC	RAD18	BRCA2	DHFR
CLASPIN	FANCD2	REV1	CHK2	EME1
RPA1	FANCE	REV3	MRE11	ERCC4
TOPBP1	FANCF	REV7	NBS1	EXO1
EP300	FANCG		RAD50	GMPS
	FANCJ		RAD51	MORF
	FANCL		RAD51B	MSL1
	FANCM		RAD51C	MUS81
			RAD51D	PARP1
			RAD52	PCNA
			RAD54L	RRM1
			RAD54L2	RRM2
			RECQ	TYMS
				TIP60

Table *S2* List of the qPCR primers for the analyzed DNA repair genes.

**Gene**	**Forward**	**Reverse**
hATR-RT	CTGAACACGGACATGTGGAC	GAAAAGGAATGGCTTGCAGA
hATRIP-RT	GAGTGCTCACGTGATGGAGA	TGTACAAGGGGGAGGAAATG
hCBP-RT	CCGGAAGATGCATCAGATTT	TCTTTTCGAGGTCTGCCAGT
hCHK1-RT	TACTGCAACCTCCGTCTCCT	AAACCTAGCCAGGTGTGGTG
hCLASPIN-RT	ATGCTTCCCAGATGGACTTG	CTCGTTCAATTCGCTCCTTC
hRPA1-RT	CATCGGTAGGCAAAGGAAAA	CTGAAGGCAGAGGGAGTGAC
hTOPBP1-RT	AAGGAGCCCTCGTTACACCT	AGTTTCCAAGGCTGCAAGTG
hEP300-RT	GATGAGAGCAACAACCAGCA	GCATGGACCAGAGACTGGAT
hFAN1-RT	CAGGGCAGGTTGGCTTAATA	TTTGTCTTTGGTGGTGGTGA
hFANCA-RT	TAGGCTCTGCTTTGCAGGAT	GATCTGTCCCACGCTAGAGG
hFANCB-RT	TGGATTCAGGTGGAGGAAAC	TTTCCCATTTAGCAGCAACC
hFANCC-RT	CACTGGCTCCAGACACTGAA	CAGGAACCAGCTCTCAAAGG
hFANCD2-RT	CCTGAGCTGCTTTTCTTGCT	CGGCTTCCTTTGTTCTTGAG
hFANCE-RT	GTGGAGATGACCCCTGAGAA	GTCAGCATGAGCTTGGCATA
hFANCF-RT	TCTGACTCAGCCTCCCAAGT	TGGTGAAACCCCGTCTCTAC
hFANCG-RT	GTACACAGCACTGGGGTCCT	CAGGCTGATCCCTCTTTCAG
hFANCJ-RT	CAGATCCACAAGCCCAACTT	CAGGTGTTGCCTTCGGTATT
hFANCL-RT	CCTGGGAATTAAGCTGAGCA	AGGATAGCACGAGCTGGAAA
hFANCM-RT	CACGAAGGGTTTTACCCAGA	ACCTTCTTCACCCACACAGG
hPOLI-RT	CCAGGGTGGGGAGTTAAAGT	CTTGCCTTTCTGGTGTCCAT
hPOLK-RT	AGGGCATTGCTTTCTCTCCT	CTCTCTCCATCCCTCGTCAG
hPOLN-RT	GCAAACCAAGAAGGTGGTGT	CTGGGCAGCTTCCTGAATAG
hRAD18-RT	GCCAGATGCTTCCAGAGTTC	ATGTGTTTGGGTTGGGTGAT
hREV1-RT	TGCTCACCACTCCACTGTTC	GTTTGGATGGCACTGAAGGT
hREV3-RT	AGCCTCATGAAGCGCATATT	GCTTTTCGGAACTTGACAGC
hREV7-RT	GTGGAGAAAGTGGTGGTGGT	CGAGTCTGAGCTGATGGACA
hATM-RT	AGGCCAAATGATTTCAGTGC	TTGGCAAACATCCATGAAGA
hBLM-RT	CGAAATCCTCCAGCATCATT	GGAGCCATAATCCCCAATTT
hBRCA1-RT	TCATGCCAGCTCATTACAGC	TAAGCCAGGCTGTTTGCTTT
hBRCA2-RT	AGGCTTCAAAAAGCACTCCA	GTGCGAAAGGGTACACAGGT
hCHK2-RT	CAAGGCTCCTCCTCACAGTC	AAGGAGCTCAGTGTCCCAGA
hMRE11-RT	CCCGTAGCAGGAACTCAAAG	GGGAAGGCTGCTGTCTTGTA
hNBS1-RT	AATGGCTTTTCCCGAACTTT	CAAGAAGAGCATGCAACCAA
hRAD50-RT	TTGGATGAGCCAACAACAAA	CCAGAAGCTGGAAGTTACGC
hRAD51-RT	GGTGAAGGAAAGGCCATGTA	CATCACTGCCAGAGAGACCA
hRAD51B-RT	AAGATGCAAACGGCTTATGG	CTTCGTCCAAAGCAGAAAGG
hRAD51C-RT	AGACGTTCCGCTTTGAAATG	GGAGTTCCTCAGCAGTCTGG
hRAD51D-RT	GCGGCAAAACTCAGGTATGT	CAGCCCTCCATTGGAATCTA
hRAD52-RT	TGGGACTGAGGAAGCAATTC	TGGCCTGGTACTCTTCTGCT
hRAD54L-RT	CTGCCTGTGAAGATTGAGCA	CCCTCAAGCAATTCTTCTGC

hRAD54L2-RT	GTTTGAACGCCCTATCCTGA	CAAAGCCCTCCAGAAGACTG
hRECQ4-RT	GGGTGCTTGTGGAGTTCAGT	CACACGGCCATAGAGGAAGT
hADSS-RT	TGGTGCCTTTCCTACAGAGC	CCAACCAGCCACATCTTCTT
hAPEX1-RT	CAAACCTGCCACACTCAAGA	CTGGGGCTTCTTCCTTTACC
hCTPS-RT	TCTACCGAGTCCCCTTGTTG	TTTCTTGGCTGCCTCTCAAT
hDHFR-RT	CCTGGTTCTCCATTCCTGAG	CTCCTTGTGGAGGTTCCTTG
hEME1-RT	TGTCAGCTTCCAGCCTACCT	CTCGGCTTGGTTTTCTTCTG
hERCC4-RT	CTGGAACTGCTCGACACTGA	GCAGTGCAGCTGGAGAAAGT
hEXO1-RT	AGCTTGGGGATGTATTCACG	TGCCTTTGCTAATCCAATCC
hGMPS-RT	CTGGATGCTGGTGCTCAGTA	AAATGCTGGTGTTTCCAAGG
hMORF-RT	GACCGCAGTACAGGGTCAAT	TCATGGGGTAGAAGGCTGAC
hMSL1-RT	CCCATCACCGTTACCATTACG	GGAACAGCCAAGACTGAAGTTT
hMUS81-RT	AGAAGTTGGCCGAGTCAGAA	AAGCTCTGCTGAAGCTGCTC
hPARP1-RT	GGAAGCTGGAGGAGTGACAG	CTTGGCATACTCTGCTGCAA
hPCNA-RT	AGGTGTTGGAGGCACTCAAG	CAAAGAGACGTGGGACGAGT
hRRM1-RT	CCTGGGGCATTTGCTATTTA	GCTGCTCTTCCTTTCCTGTG
hRRM2-RT	CTGGCTCAAGAAACGAGGAC	TCAGGCAAGCAAAATCACAG
hTYMS-RT	CCAAAGCTCAGGATTCTTCG	AGTTGGATGCGGATTGTACC
hTIP60-RT	AATGTGGCCTGCATCCTAAC	TGTTTTCCCTTCCACTTTGG

Table ***S3*** List of shRNA target sites of indicated genes.

**Gene**	**Hairpin sequences**
Tip60-1	ACCGAAAGCTTTTCTATGTCC
Tip60-2	CCCCAACCACCGCTCAACGAA
Tip60-3	ATACAAGGGCCAGTATATCCT
Tip60-4	CACCGAAGTTACTGGTCCCAA
Cbp-1	AAGGGATGAATACTATCATTT
Cbp-2	CATGGACCTTTCTACCATCAA
Cbp-3	AAAGGCGTGTATACATATCTT
Cbp-4	ACCCAAGTGAAGGAGATGACT
Myst4-1	CCCCATGGGTTGGACAAGAAA
Myst4-2	CAAGCTGTACATTTTAACATA
Myst4-3	ACACGGACTTTAACGATGCAA
Myst4-4	CCACACAGACCAAAGTCCTCA
Msl1-1	ACAGGTTGGAGATCCAGAAGA
Msl1-2	CCAGCGAGGATGTAGAGAGAA
Ml1-3	CCCCCATTTGGAAATACAGAA
Msl1-4	ACAGAGTTGTCTGAGAAAATT

Table *S4* List of qPCR primers used for analyzing shRNA knockdown efficiency.

Gene	Forward	Reverse
MYST4-RT-1	GGAAGAGCGAGAAGCTGAGA	TTGCAGACGATTGCCTACTG
MYST4-RT-2	GTGCTTTTCCGTCCTCACTCC	CACGATTTGACTCTTTAGTCCCC
MSL1-RT-1	TGCTGATTACCCCCTTCTTG	CTACAGGTCCGGTGAGGTGT
MSL1-RT-2	TCCCCAACTGGGCAGAATC	GACAGCGGTGATGATAACAGG
TIP60-RT-1	TCCCGGTCCAGATCACACTC	ACCTTCCGTTTCGTTGAGCG
TIP60-RT-2	GGGGAGATAATCGAGGGCTG	TCATCCAGACGTTTGTTGAAGTC
CREBBP-RT-1	GGCTTCTCCGCGAATGACAA	GTTTGGACGCAGCATCTGGA
GAPDH-RT	CCTGGAGAAACCTGCCAAGTATG	AGAGTGGGAGTTGCTGTTGAAGTC

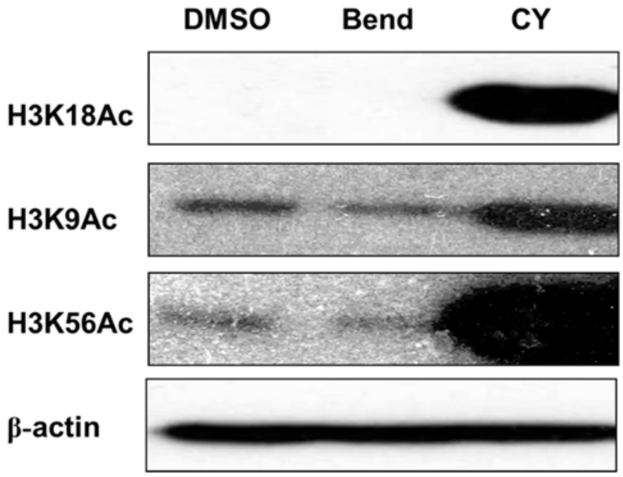

Figure *S1* HDAC inhibition by CY and Cpd A, but not bendamustine in H1650 cells. The HDAC inhibitor SAHA were used as positive control. Cells were treated at indicated drug concentration for 8hrs before collecting samples.

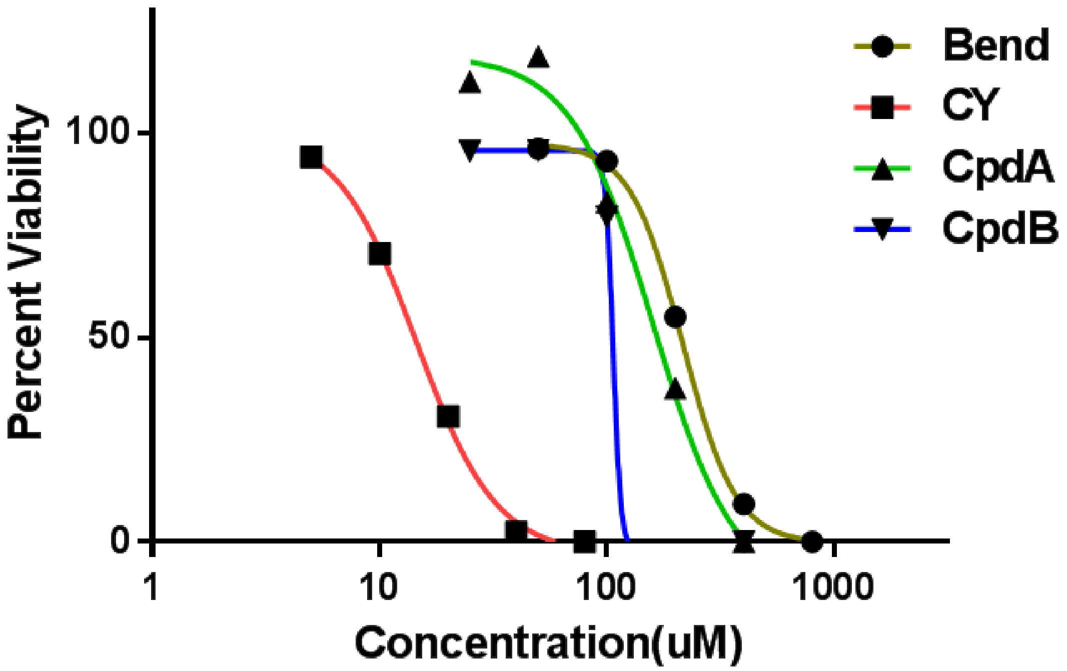

Figure ***S2*** Dose response curve of bendamustine, CY, Cpd A and Cpd B in H1650 cells. Cells were treated for 48hrs before MTT assay.

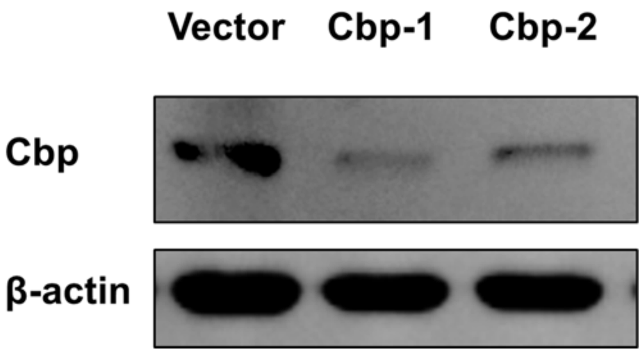

Figure *S3* ShRNA mediated suppression of Cbp in Myc Arf-/- cells at protein level.

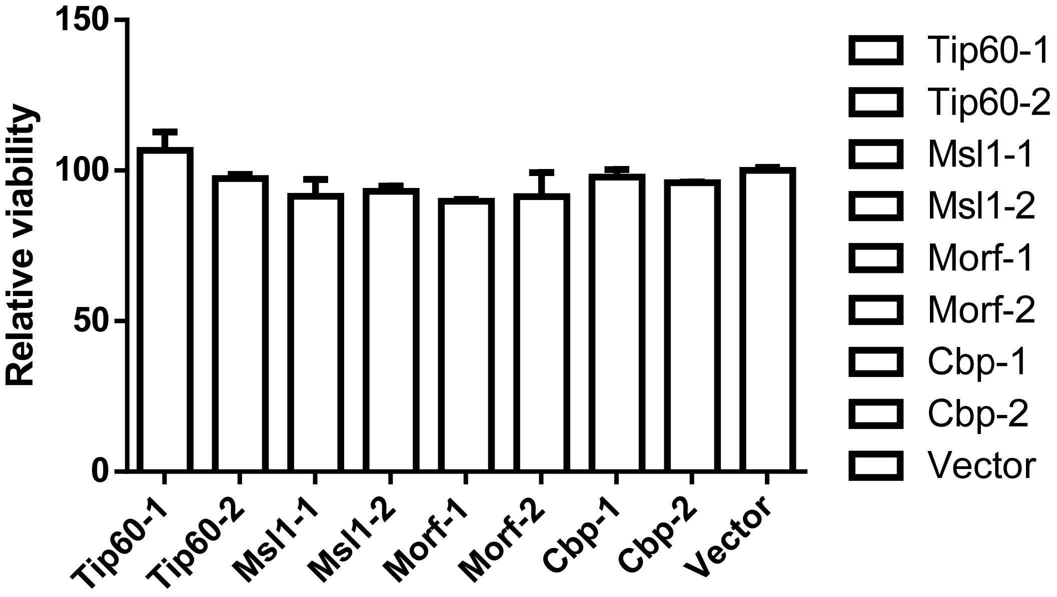

Figure ***S4*** Suppression of CBP, Tip60, MORF and MSL1 don't induce cell death without drug treatment. GFP percentage of Cells infected with shRNA were examined for at least 72h, and relative viability were calculated by similar formula for relative resistance index. Data represent mean±SEM, from at least two independent experiments. All shRNA groups show no significant different from vector control.

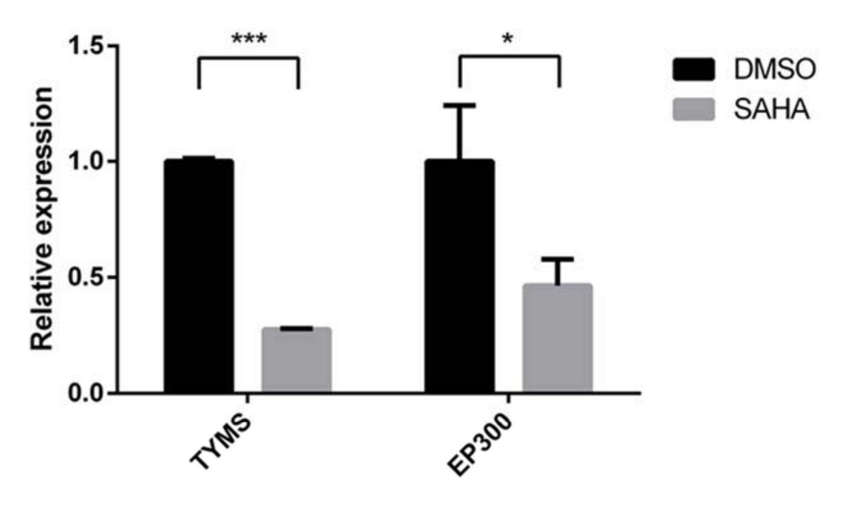

Figure *S5* HDAC inhibition resulted in suppression of EP300 and TYMS. Cells were treated for 6hrs before mRNA collection and qPCR analysis. Data represent mean±SEM, from three independent experiments. $*p < 0.1$, $**p < 0.05$, $***p < 0.01$.

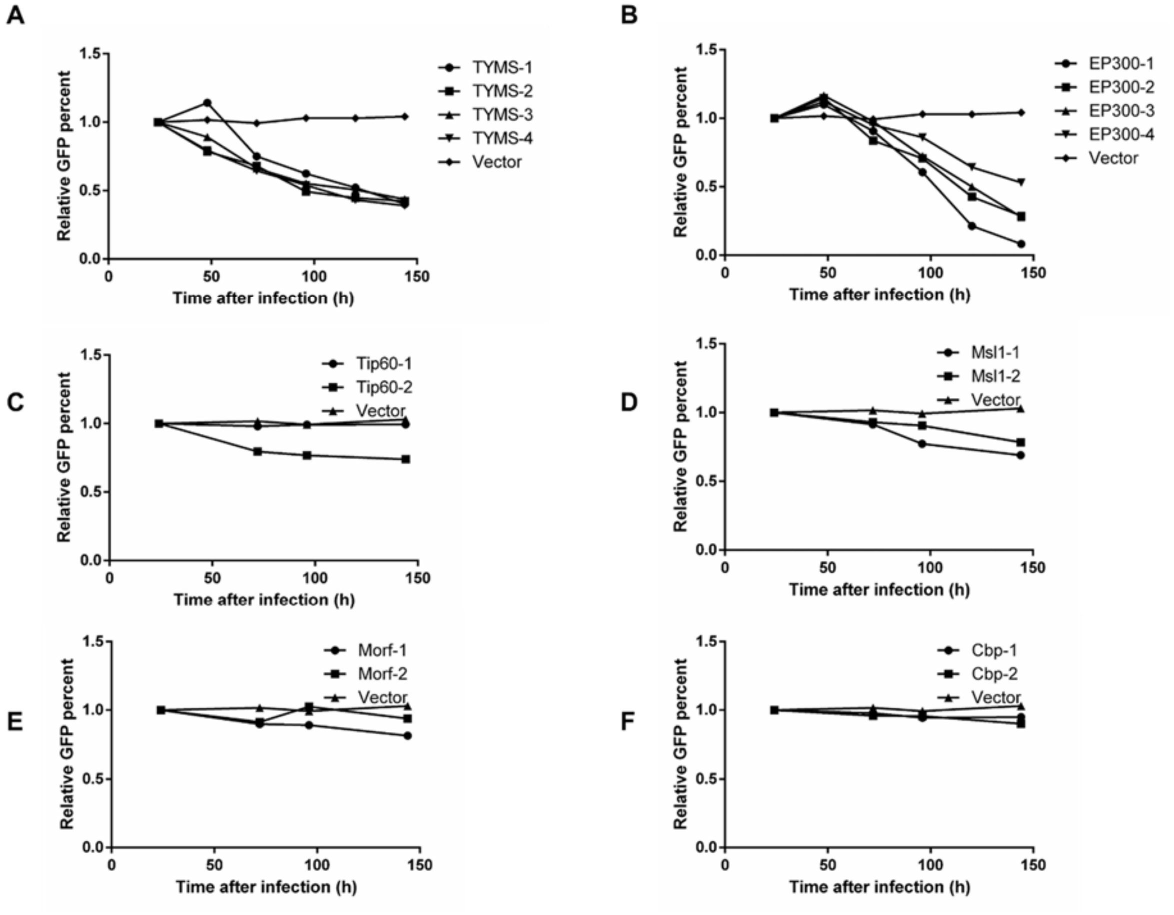

Figure *S6* Suppression of EP300 and TYMS caused reduced viability of cancer cells. Retrovirus that encodes GFP and indicated shRNAs were used to infect cells to achieve 30-60% infection rate, and percentages of GFP positive, shRNA-expressing cells were followed overtime. shRNAs that significantly impairs cell growth such as shEP300 and shTYMS showed rapid loss of GFP positive cells overtime.
