# Supplementary figures and images for: A DNA/HDAC dual-targeting drug CY190602 with significantly enhanced anticancer potency
Source: EMBO Mol Med. 2015 Mar 10;7(4):438–49. doi: 10.15252/emmm.201404580 (PMC4403045; doi:10.15252/emmm.201404580)

**Figure S3**

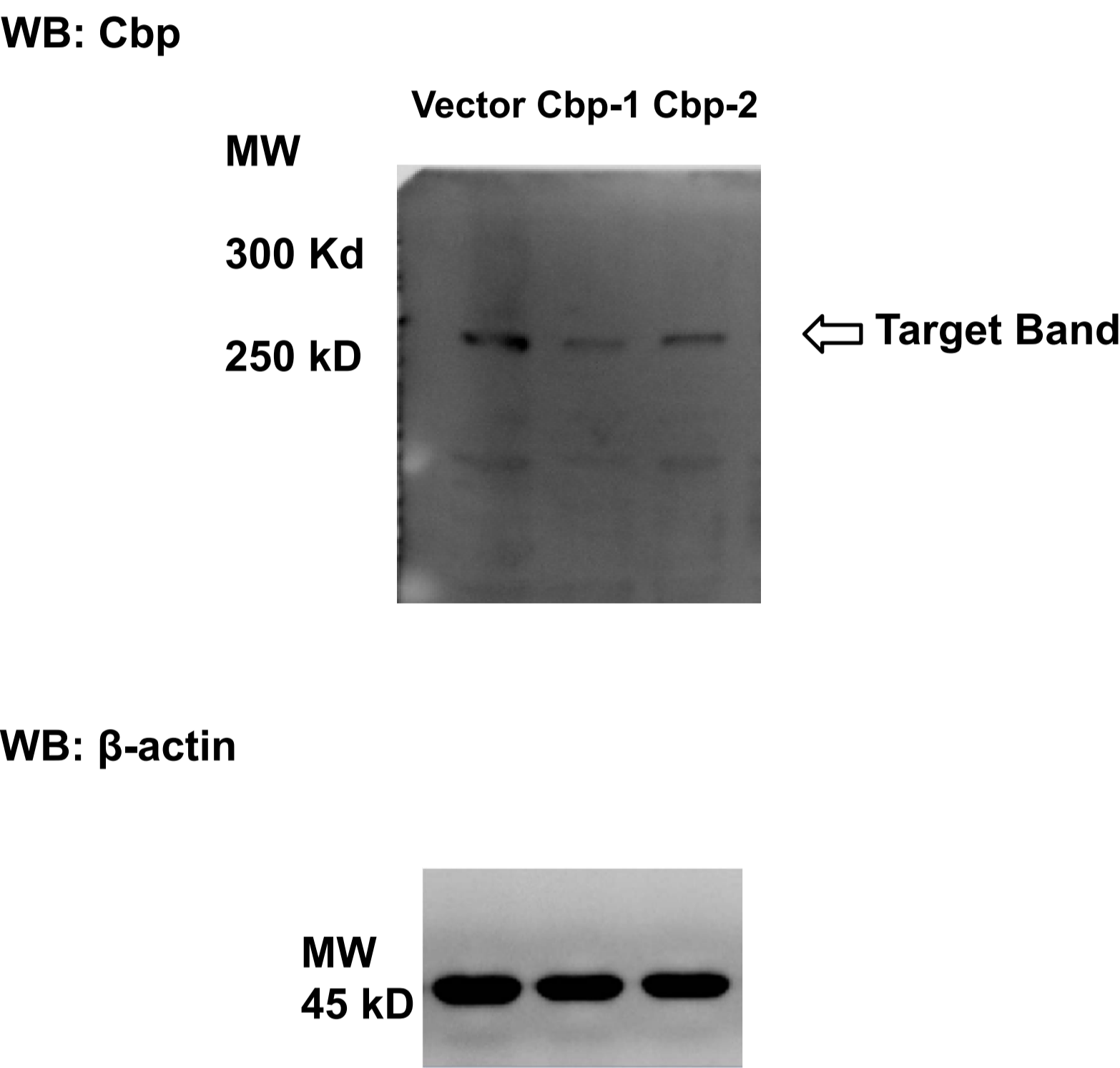

Supplement: Supplementary file 2 — Source Data for Supplementary Figure S3 [file emmm0007-0438-sd2.pdf]

Figure 1D

WB:  $\gamma$ - H2AX

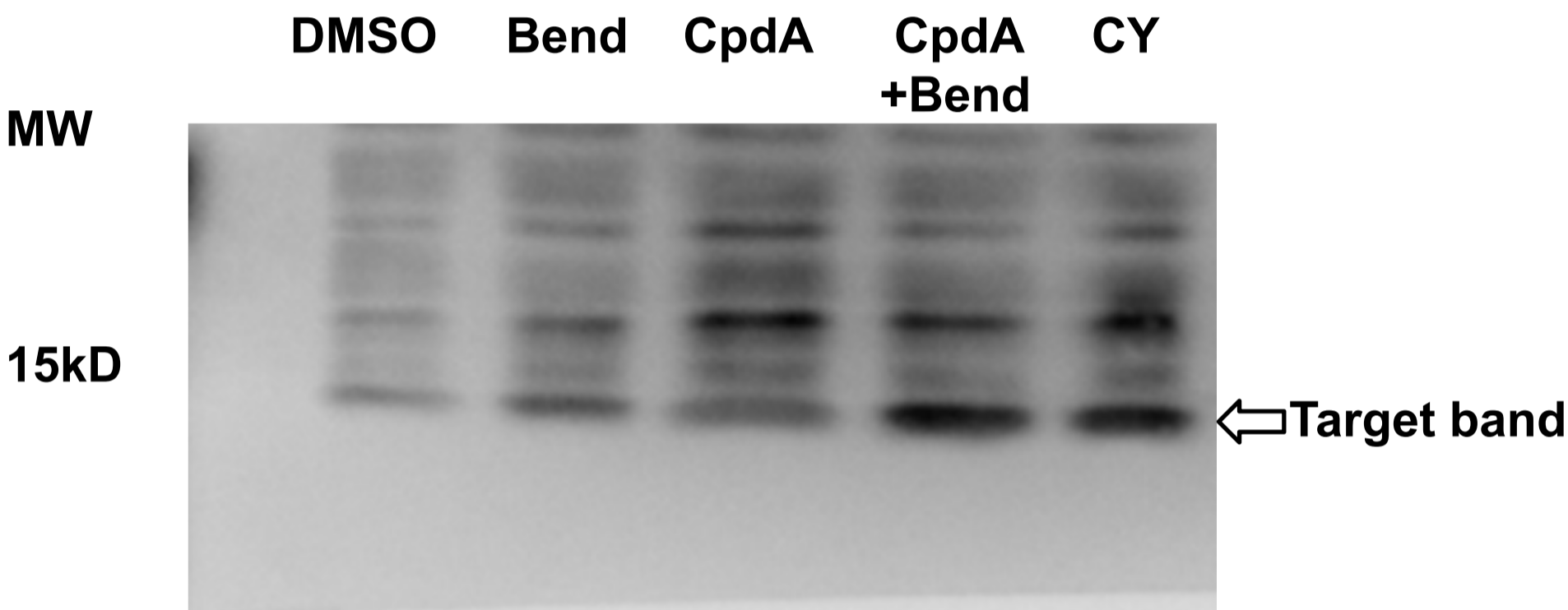

WB:  $\beta$ -actin

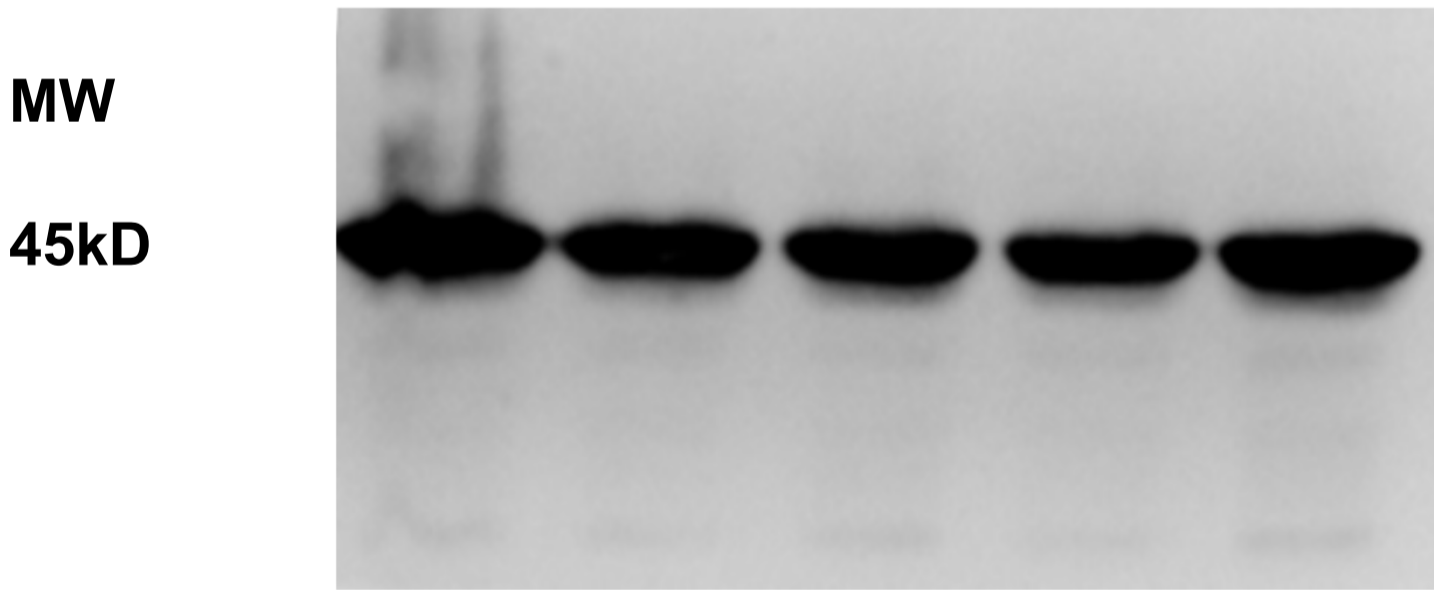

Supplement: Supplementary file 4 — Source Data for Figure 1 [file emmm0007-0438-sd4.pdf]

Figure 2B

WB:H3K18Ac

DMSO CY CpdA CpdB Bend SAHA

MW

15kD

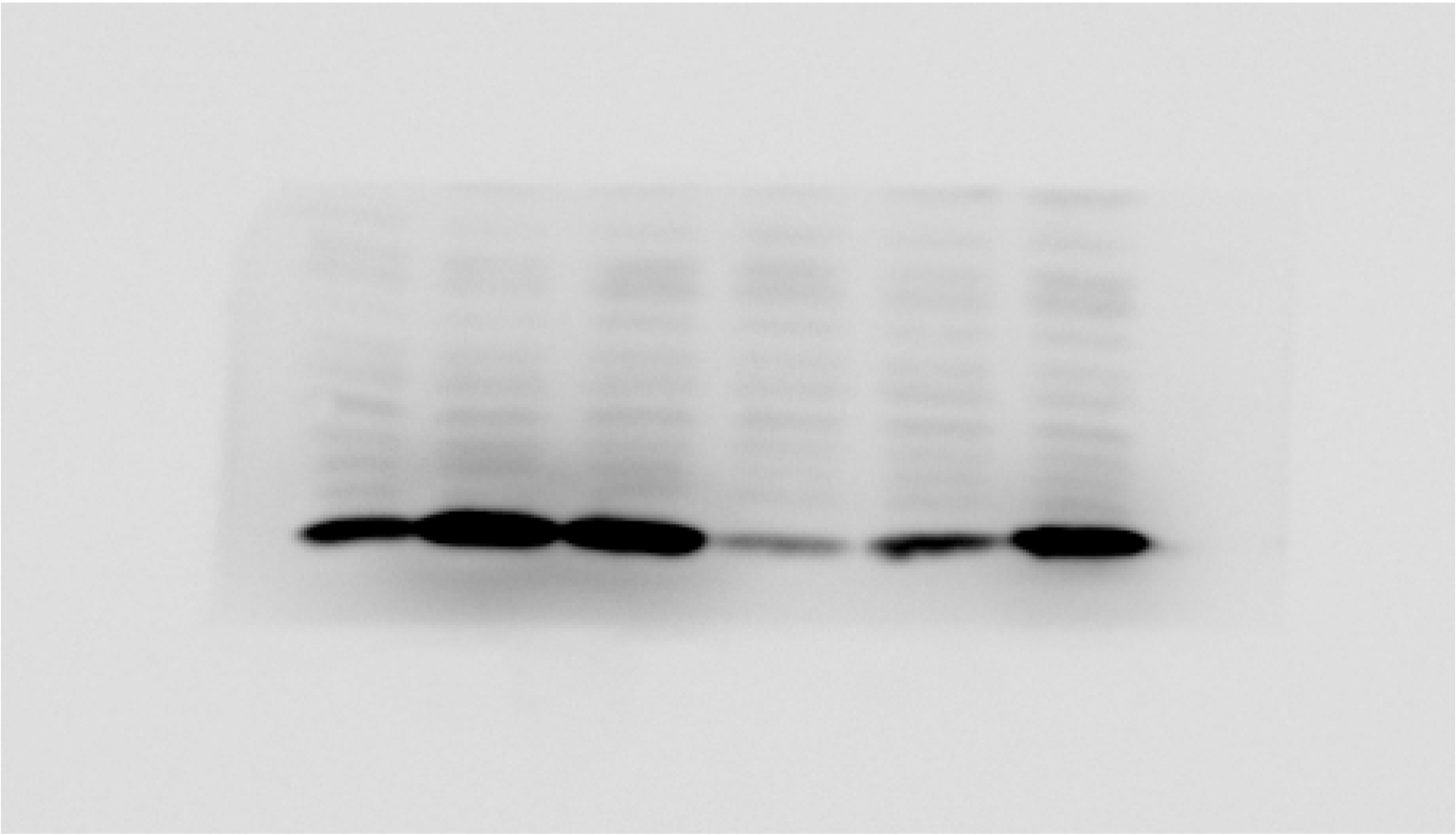

WB:  $\beta$ -actin

MW

45kD

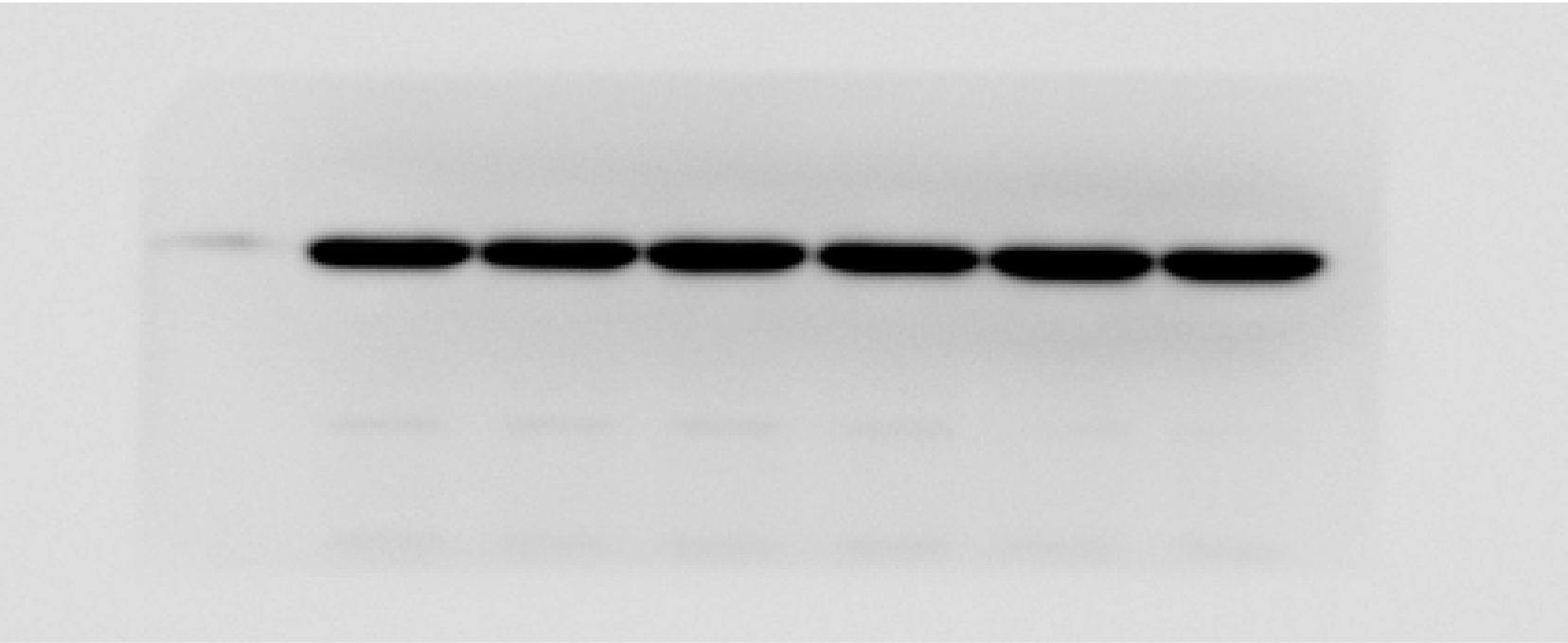

Supplement: Supplementary file 5 — Source Data for Figure 2 [file emmm0007-0438-sd5.pdf]

Figure 3B

WB: PARP

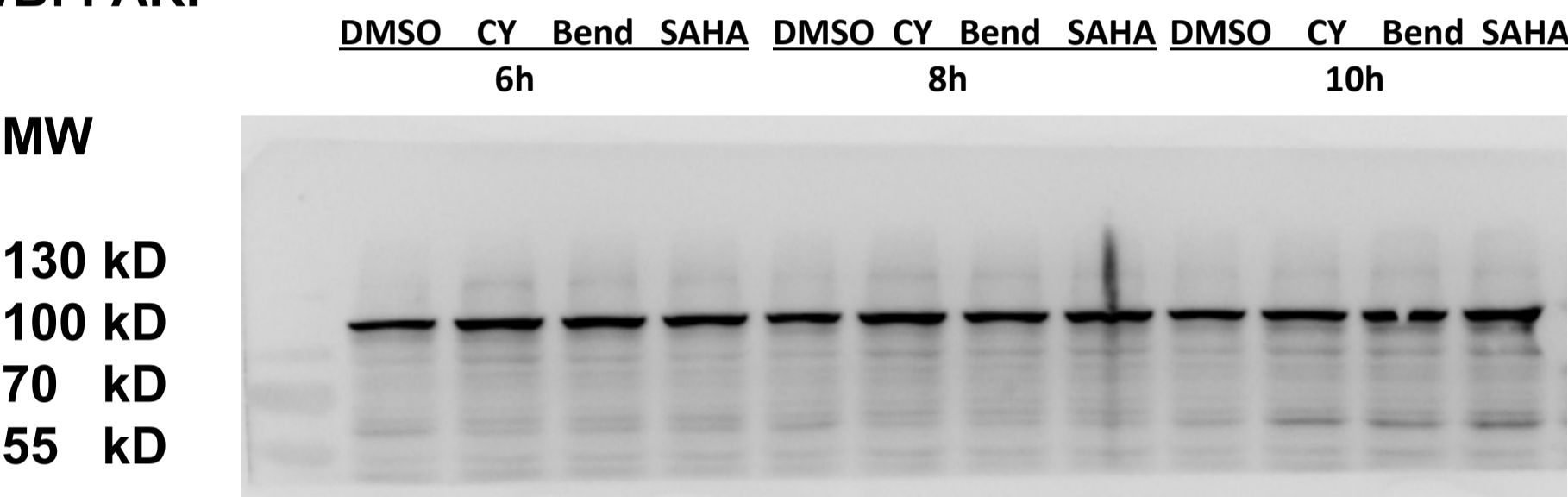

WB:  $\beta$ -actin

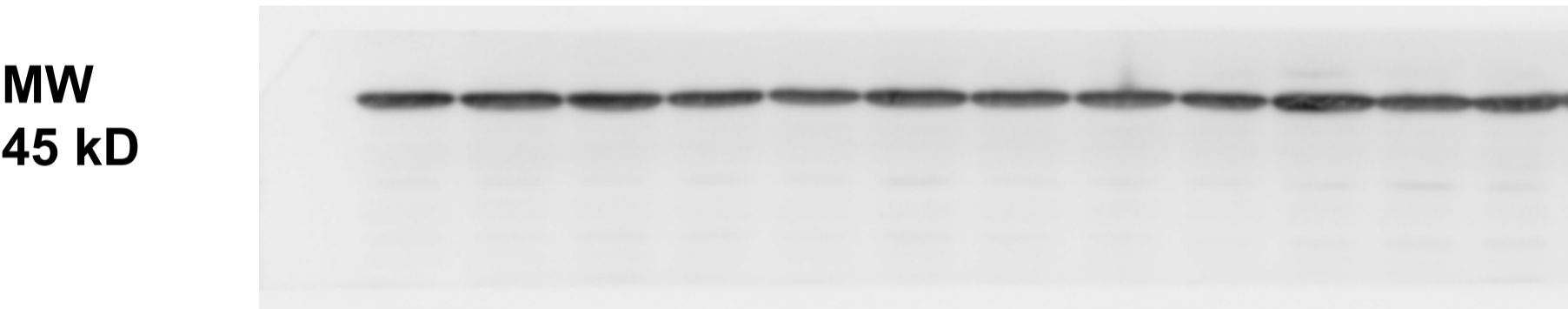

Supplement: Supplementary file 6 — Source Data for Figure 3 [file emmm0007-0438-sd6.pdf]
